# Oxidative DNA damage and oxidized low density lipoprotein in Type II diabetes mellitus among patients with *Helicobacter pylori* infection

**DOI:** 10.1186/s13098-016-0149-1

**Published:** 2016-05-03

**Authors:** Wesam Ahmed Nasif, Mohammed Hasan Mukhtar, Mohammed Mahmoud Nour Eldein, Sami Sadagah Ashgar

**Affiliations:** Biochemistry Department, Faculty of Medicine, Umm Al-Qura University, Makkah, Kingdom of Saudi Arabia; Molecular Biology Department, Genetic Engineering and Biotechnology Research Institute, Sadat City University, Sadat City, Egypt; Oncology Diagnostic Unit, Faculty of Medicine, Ain Shams University, Cairo, Egypt; Microbiology Department, Faculty of Medicine, Umm Al-Qura University, Makkah, Kingdom of Saudi Arabia

**Keywords:** *Helicobacter pylori*, Type 2 diabetes mellitus, 8-Hydroxydeoxyguanosine, Oxidized low density lipoprotein

## Abstract

**Background:**

*Helicobacter pylori* (*H. pylori*) infection is reported to be associated with various extragastrointestinal conditions such as insulin resistance, diabetes mellitus and metabolic syndrome. *H*. *pylori* infection and type 2 diabetes mellitus (T2DM) are associated with oxidative stress, this cross-relation between *H. pylori* induced infection in T2DM and oxidative damage is still debated. Thus, the question arises whether an increase in the serum level of 8-OHdG and Ox-LDL will occurs in patients with T2DM infected *H. pylori*; this will be through determination and compare frequency of *H. pylori* infection in T2DM and non-diabetic patients.

**Methods:**

100 patients presented with history of epigastric discomfort for more than 1 month; 50 patients with T2DM and 50 non-diabetics. Anti-*H. pylori* IgG using ELISA, fasting and postprandial glucose level, glycated hemoglobin (HbA1c) and body mass index (BMI) was calculated. Serum 8-OHdG and Ox-LDL was measured using ELISA for the 100 patients and 50 control subject.

**Results:**

Rates of *H. pylori* infection of T2DM and non-diabetic were 66 and 58 %, respectively, (*p* = *0.001*). *H. pylori IgG* antibody was not correlated with HbA1c either in T2DM (*p* = *0.06*) or non-diabetic (*p* = *0.25*). Serum 8-OHdG level in T2DM with positive *H. pylori* infection showed a significant difference compared to non-diabetics with positive *H. pylori* infection (*p* = *0.001*) and higher than that in T2DM with negative *H. pylori.* A correlation between 8-OHdG concentration and HbA1c in T2DM patients infected with *H. pylori* was observed (r = 0.39, *p* = *0.02*). Serum Ox-LDL level in T2DM with positive *H. pylori* infection showed a significant difference compared to diabetics with both negative *H. pylori* infection and in non-diabetics with positive *H. pylori* infection (*p* = *0.001*).

**Conclusions:**

Increased levels of oxidative DNA damage (8-OHdG) and Ox-LDL suggest the mechanistic link between *H. pylori* infection combined with diabetes and increased generation of ROS and could play as an important image for high risk to atherosclerosis.

## Background

*Helicobacter pylori* (*H. pylori*) infection is probably one of the most common chronic bacterial infections worldwide [1]. The stomach is the primary site of *H. pylori* infection, including chronic active gastritis, peptic ulcer, gastric adenocarcinoma and type B low-grade mucosa-associated lymphoid tissue lymphoma [2]. *H. Pylori* is responsible for both gastric local inflammation and a systemic inflammation leading to extra-gastrointestinal tract conditions such as cardiovascular diseases, idiophatic thrombocytophenic purpurae (ITP), unexplained iron deficiency anemia, diabetes mellitus (DM) and insulin resistance [3]. Different studies reported that prevalence of chronic *H. pylori* infection associated with both gastrointestinal and extra-intestinal ailments [4–6].

Gastrointestinal inflammation caused by *H. pylori* can influence the absorption of glucose and lipids, which are also abnormal in diabetes mellitus [6]. Diabetes has been identified as a risk factor for an extragastric manifestation of *H. pylori* infection [7, 8]. The relationship between *H. pylori* and DM was first explored in 1989 by Simon et al. [9] who found that the prevalence of *H. pylori* infection in patients with DM was significantly higher than in asymptomatic controls (62 vs 21 %). The link between *H. pylori* infection and diabetes remains controversial, as some studies indicated a higher prevalence of infection in diabetic patients [10, 11], while others reported no difference [12, 13].

Recent evidence implicates the pathological involvement of inflammation in type 2 diabetes mellitus (T2DM), which is an important process induced by *H. pylori* infection [14]. As insulin resistance can be developed in the presence of inflammation [15] or as a result of alterations in counter regulatory hormones that affect insulin [16]. Thus, *H. pylori* may promote insulin resistance by inducing chronic inflammation and affecting insulin-regulating gastrointestinal hormones as reported by Aydemir et al. [17].

Furthermore, *H. pylori* infection is strongly linked to the pathogenesis of T2DM, which is associated with a general activation of the innate immune system, and a chronic, cytokine-mediated state of low-grade inflammation [14]. The host immune response to *H. pylori* infection is complex and involves up regulation of several pro-inflammatory cytokines, such as C-reactive protein (CRP) [18], interleukin 6 (IL-6), and tumor necrosis factor- α (TNF-α) [19], which are implicated in insulin resistance and the development of diabetes [20]. Thus, a potential relationship between *H. pylori* infection and diabetes is highly suspected. For this reason; it is expected in our study that, the implication between *H. pylori* and diabetes induces inflammation, accumulation of reactive oxygen species (ROS) and oxidative DNA damage in gastric mucosa.

Oxidative stress due to overproduction of ROS through either endogenous or exogenous insults can damage cellular macromolecules, leading to DNA damage, protein modification, lipid peroxidation and associated with the development of several diseases such as cardiovascular, chronic inflammation and cancer [21]. ROS production has been reported to be increased in DM [22] and *H. pylori* infection [5] which may directly contribute to the generation of oxidative stress via several mechanisms.

*H. pylori* infection induces infiltration and activation of neutrophils and macrophages [23]. Enhanced ROS levels due to neutrophil infiltration and increased oxidative DNA damage have been reported in *H. pylori*-infected patients [23]. Moreover, neutrophil accumulation and oxidative stress at sites of *H. pylori*-mediated gastric inflammation induce enhanced localization of Ox-LDL at such gastric inflammatory lesions and increased plasma levels of Ox-LDL occur in patients with *H. pylori*-positive chronic gastritis [24].

On the other hand, oxidative stress can be induced in hyperglycemia status via glucose autoxidation and the formation of advanced glycation end-products (AGE) [25]. Other circulating factors that are elevated in diabetics, such as free fatty acids and leptin, also contribute to increased ROS generation [26]. Furthermore, oxidative stress has been increasingly implicated in the deterioration of pancreatic islet function [27].

Ox-LDL has been observed to be increased in diabetic patients and this may contribute to the increased atherogenesis in diabetes, regardless of normal lipid levels, Ox-LDL levels may be elevated in diabetic patients and this may be the explanation for the altered endothelial function [28]. Endothelium exposed to Ox-LDL develops alterations such as endothelial damage. The Ox-LDL itself activates inflammatory cells and potentiates the liberation of growth factors from monocytes/macrophage [29].

ROS attack guanine bases in DNA easily and form 8-hydroxydeoxy guanosine (8-OHdG), which can bind to thymidine rather than cytosine, based on which, the level of 8-OHdG is generally regarded as a biomarker of mutagenesis consequent to oxidative stress, and as a risk factor for many diseases including *H. pylori* infection [30] and diabetes [31]. A recent study reported an increase in the 8-OHdG content in mononuclear cells and ROS level in Type I (insulin-dependent) and Type II (non-insulin-dependent) diabetic patients when compared with control subjects [32]. Another study reported that *H. pylori*-induced chronic inflammation and oxidative stress create an environment conducive to DNA damage and tissue injury [33].

The previous reported studies were based on studying the role of oxidative stress in *H. pylori* and diabetes separately. However, to our knowledge, the association between diabetes and oxidative status has not been previously investigated in *H. pylori* infection. Therefore, the goal of this study was to assess the prevalence of *H. pylori* infection in patients with T2DM and to evaluate the relationship between the level of oxidative DNA damage 8-OHdG and serum Ox-LDL levels in T2DM patients with *H. pylori* infection.

## Methods

### Subjects

This study was conducted in 100 patients with history of dyspepsia or epigastric discomfort for more than 1 month and where known as cases of T2DM for approximately 5 years duration. This study was done in collaboration between biochemistry department, faculty of medicine, Umm Al-Qura University, Makkah Al-Mukarama, Kingdom of Saudi Arabia and Sadat City University, Sadat City, Egypt. Patients with the following conditions were excluded from the study: Patients of type-I diabetes, pregnancy, prior *H. pylori* eradication therapy, use of antibiotics therapy, history of gastric surgery or cholecystectomy, chronic renal failure requiring dialysis treatment and patients who were diagnosed for malignancy.

The inclusion criteria of the study was investigated for T2DM and *H. pylori* infection, and divided into two groups- A and B, as well as a 50 healthy control group. Group-A (labeled diabetic group) contains 50 diabetic patients known cases of T2DM with positive or negative *H. pylori* infection (40 females, 10 males, mean age 51.04 ± 7.4 years); while group-B (labeled non diabetic group) contains 50 non diabetic patients with positive or negative *H. pylori* infection (19 females, 31 males, mean age 43.3 ± 9.3 years). The known cases of DM in group-A were also investigated for blood sugar levels (not for diagnostic purpose but to assess the blood sugar level that whether it is controlled or uncontrolled). Height and body weight were measured using a digital scale, and body mass index (BMI) was calculated as follows: BMI = body weight (kg)/height squared (m^2^). The study protocol was approved by Ethics Review Board for Human Studies at Faculty of Medicine, Umm Al-Qurra University and conformed to the ethical guidelines of the 1975 Helsinki declaration.

### Samples and laboratory methods

Blood samples were obtained following an overnight fasting period according to the diagnostic criteria of DM; only patients with fasting blood glucose above 126 mg/dL or postprandial 2 h after meal above 200 mg/dL. Samples were withdrawn from a cubital vein into blood tubes and immediately serum was separated from the cells by centrifugation at 3000 r/min for 10 min and stored in refrigerator at 4 °C until processed. Fasting blood sugar (FBS) level and postprandial blood sugar (PBS) level were measured using an auto analyzer (COBAS INTEGRA 400 PLUS, Roche, Germany). In addition, hemoglobinA1c (HbA1c) was measured according to DCCT (diabetes control and complications trial), as well as rapid urease test was determined to check the presence of *H. pylori*.

### Quantitative determination of anti-H. Pylori IgG

*H. pylori* status was defined by the titer of *H. pylori* antibody using *H. pylori* IgG enzyme-linked immunosorbent assays (ELISA) (Ratio Diagnostics, Frankfurt, Germany) for the detection and qualitative determination of IgG antibodies to *H. pylori* in human serum. A value <0.9 is considered negative for the presence of detectable IgG antibody and values greater than 1.1 indicated the presence of detectable IgG antibody against *H. pylori*.

### Determination of serum 8-Hydroxydeoxyguanosine (8-OHdG)

8-OHdG was measured using the commercially available Cloud-Clone Crop 8-OHdG Competitive inhibition enzyme immunoassay technique kit (Cloud-Clone Crop., USCN Life science Inc.; Houston, TX 77,082, USA), intended to be used for the in vitro quantitative measurement of 8-OHdG in human blood serum. A monoclonal antibody specific to 8-OHdG has been pre-coated onto a micro plate. A competitive inhibition reaction was then launched between biotin labeled 8-OHdG and unlabeled 8-OHdG (standards or samples) with the pre-coated antibody specific to 8-OHdG. After incubation the unbound conjugate is washed off. Avidin conjugated to horseradish peroxidase (HRP) was then added to each micro plate well and incubated for 30 min at 37 °C. The amount of bound HRP conjugate was reverse proportional to the concentration of 8-OHdG in the sample. After addition of the substrate solution and the absorbance was measured with a micro-plate reader at a wavelength of 450 nm, the intensity of color developed was reversed proportional to the concentration of 8-OHdG in the sample. The amount of 8-OHdG in the serum was calculated from the standard curve and results were expressed as pg/ml.

### Determination of oxidized low density lipoproteins (Ox-LDL)

Ox-LDL was measured using the commercially available Cloud-Clone Crop Ox-LDL Competitive sandwich enzyme immunoassay technique kit (Cloud-Clone Crop., USCN Life science Inc.; Houston, TX 77082, USA), intended to be used for quantitative measurement of Ox-LDL in human blood serum. The micro titer plate provided in this kit has been pre-coated with an antibody specific to Ox-LDL. Standards and samples were then added to the appropriate micro titer plate wells with a biotin-conjugated antibody specific to Ox-LDL. Avidin conjugated to HRP was then added to each micro plate well and incubated for 30 min at 37 °C. After 3,3′,5,5′-Tetramethylbenzidine (TMB) substrate solution is added, only those wells that contain Ox-LDL, biotin-conjugated antibody and enzyme-conjugated avidin exhibited a change in color. The enzyme-substrate reaction terminated by the addition of sulphuric acid solution and the color change was assessed spectrophotometrically at a wavelength of 450 nm. The concentration of Ox-LDL in the samples was then determined by comparing the O.D. of the samples to the standard curve.

### Statistical analysis

All statistical analyses were done using a Statistical Package for the Social Sciences (SPSS); v.20 (SPSS Inc., Chicago, IL, USA). Continuous variables were expressed as mean ± SD, whereas categorical variables were expressed as numbers (percentages). Statistically significant differences between groups were determined using Student *t* test and Manne Whitney U-test. Statistical comparisons between more than three groups such as data relating to circadian variation of Ox-LDL and 8-OHdG were performed by 1-way ANOVA. The correlation coefficient was obtained by Pearson correlation test. *P* values less than 0.05 were considered to be significant.

## Results

### Baseline characteristics

Among 100 patients in our study, fifty patients in each group, of which 41 (41 %) males and 59 (59 %) females. The overall mean age was 47.17 ± 9.2 years. The mean age of male’s 46.34 ± 9.52 and 48.37 ± 8.91 years old in female patients respectively. The prevalence of *H. pylori* infection among T2DM and non-diabetics patients was 66 % (33/50, mean age 51.79 ± 6.9 years) and 58 % (29/50, mean age 44.86 ± 10.08 years) respectively. This was statistically significant (*p* = *0.001*) (Table 1).

**Table 1:** Demographic and biochemical characteristic in diabetic and non- diabetic patients with (+Ve) *H. pylori* and (−Ve) *H. pylori* infection

Mean ± SD						
	Healthy (n = 50)	Non-diabetic (n = 50)		Diabetic (n = 50)		*P* value^*^
	−ve *H. pylori*	−ve *H. Pylori* (n = 21)	+ve *H. Pylori* (n = 29)^b^	−ve *H. Pylori* (n = 17)	+ve *H. Pylori* (n = 33)^b^	
M/F	41/9	7/10	3/30	12/9	19/10	<0.05^*^
Age (years)	27.18 ± 4.39	41.14 ± 7.95	44.86 ± 10.08	49.59 ± 8.47	51.79 ± 6.95	<0.0001^*^
BMI (Kg/m^2^)	25.52 ± 1.11	25.86 ± 1.68	27.72 ± 2.22	28.53 ± 2.24	28.88 ± 2.68	>0.05
F.B.S (mg/dl)^a^	78.64 ± 5.15	81.81 ± 7.08	82.79 ± 8.00	187.41 ± 57.32	182.91 ± 57.55	<0.0001^*^
2 h.PP.B.S (mg/dl)^a^	93.14 ± 6.99	101.67 ± 7.96	101.10 ± 12.39	341.76 ± 67.14	321.06 ± 79.81	<0.0001^*^
HbA1C %^a^	3.39 ± 0.49	3.84 ± 0.70	3.97 ± 0.52	7.30 ± 0.55	6.93 ± 0.69	<0.001^*^
*H. Pylori* IgG	0.86 ± 0.52	3.23 ± 1.73	66.01 ± 17.36	2.46 ± 2.20	94.52 ± 45.9	<0.001^*^

* *p* < *0.05* is considered significant

^a^ References values: fasting blood glucose (F.B.S) 80–120 mg/dl; 2-hours postprandial blood glucose (2 h.pp.B.S)110–140 mg/dl; hemoglobin A1c (HbA1c) 3–6.5 %

^b^ The prevalence of *H. pylori* infection among diabetic and non-diabetics patients was 66 and 58 % (*P* = *0.001*). Positive (+*Ve*) *H. pylori* infection and negative (−*Ve*) *H. pylori* infection

Demographic characteristic are shown in Table 1. Body mass index (BMI), the serum glucose level and HbA1c were found to be significantly higher in T2DM patients than in those non-diabetic patients (*p* = *0.0001*). In addition, positive *H. pylori* in T2DM patients was higher in HbA1c 6.93 ± 0.69 when compared with positive *H. pylori* in non-diabetic patients 3.97 ± 0.52 (*p* = *0.001*). Moreover, BMI was higher in T2DM patients with *H. pylori* infection than in those without, although it did not reach statistical significance (28.88 ± 2.68 vs 27.72 ± 2.22 kg/m^2^, *p* = > *0.05*).

### Qualitative analysis of H. pylori IgG antibody in diabetic and non-diabetic patients

**Fig. 1:**
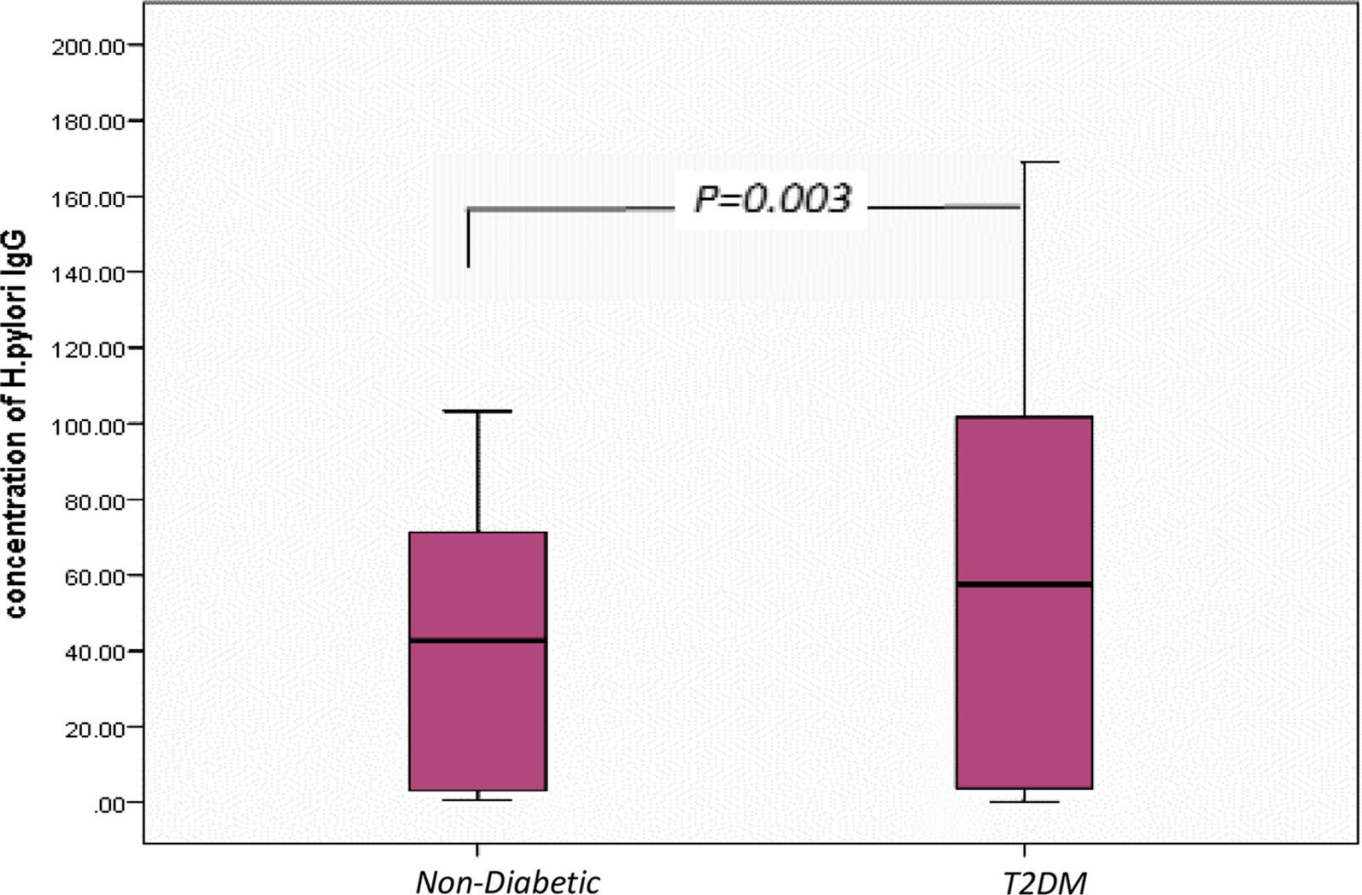
*Box plot* for *H. pylori* IgG concentration in non-diabetic and diabetic patients. The *box* represents the interquartile range. The *whiskers* indicate the highest and lowest values, and the *line* across the *box* indicates the median value. Overall significance of differences between non-diabetic and diabetic group was determined by 1-way ANOVA

IgG antibodies have been the most specific class of antibodies for the detection of *H. pylori* infection, and therefore, we analyzed IgG antibody for confirmation of *H. pylori* infection in 100 patients and 50 control. The mean ± SD of *H. pylori IgG* antibody in T2DM was 63.32 ± 57.66; in non-diabetics was 39.5 ± 33.90. There was a significant difference observed between serum *H. pylori IgG* antibody in T2DM patients compared to non-diabetics (*p* = *0.003*), shown in Fig. 1. Furthermore, positive *H. pylori* infection in T2DM patients had the highest concentration of *H. pylori IgG* antibody 94.52 ± 45.9 compared to non-diabetics 66.01 ± 17.36 and control 0.86 ± 0.52, Significant difference was observed (*p* < *0.001*) (Table 1).

**Fig. 2:**
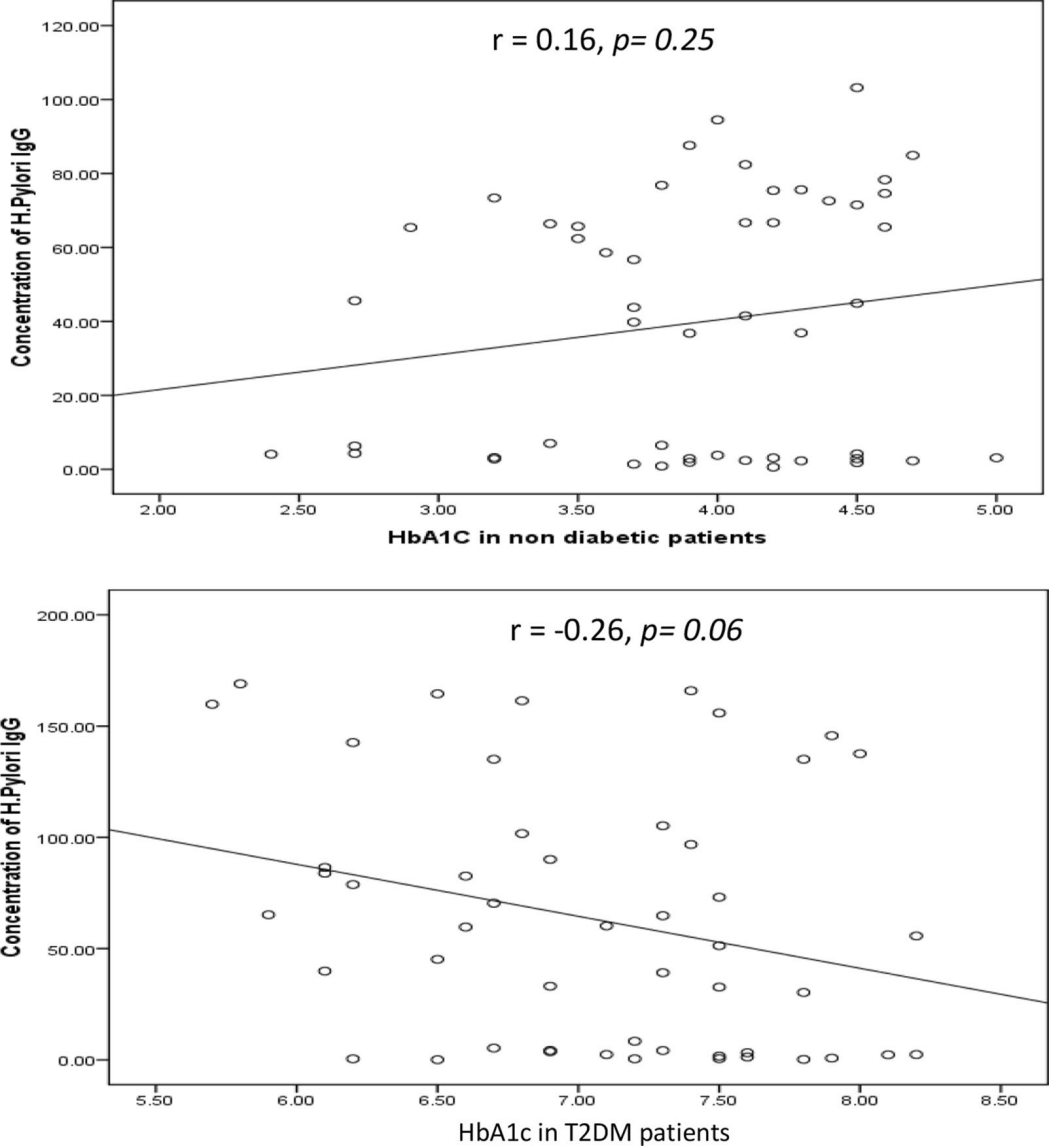
Correlation between HbA1c in non-diabetic and diabetic patients measured by ELISA (expressed in %) and concentration of *H. pylori* IgG (expressed in U). The levels of *H. pylori* IgG (*y axis*) were correlated with those of  % HbA1c (*x axis*). No association was observed in two groups. Based on the simple linear regression of cases with HbA1c (n = 50 for each group)

The correlation between the levels of the *H. pylori IgG* antibody and HbA1c is shown in Fig. 2. *H. pylori IgG* antibody was not correlated with HbA1c either in T2DM (Pearson correlation coefficient (r) = −0.26, *p* = *0.06*) or in non-diabetic patients (r = 0.16, *p* = *0.25*). Based on the simple linear regression of cases with and without diabetes (n = 50).

### Distribution of serum 8-OHdG as an oxidative DNA damage

**Fig. 3:**
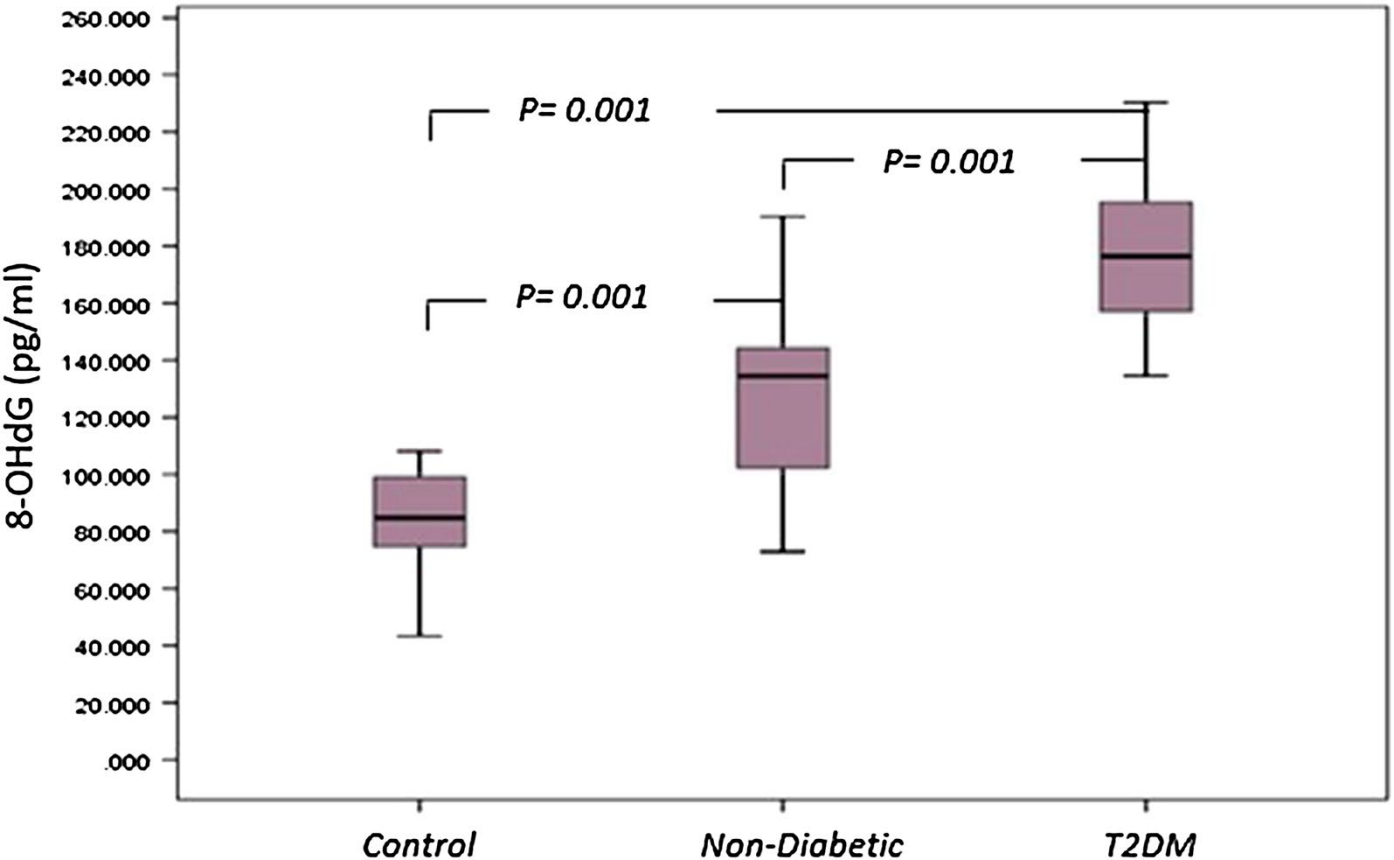
*Box plot* for 8-OHdG as an indicator of oxidative damage of DNA in non-diabetic, diabetic patients and control. The *box* represents the interquartile range. The *whiskers* indicate the highest and lowest values, and the *line* across the *box* indicates the median value. Overall significance of differences between non-diabetic and diabetic group was determined by 1-way ANOVA

8-OHdG was measured as an indicator of oxidative damage of DNA. 8-OHdG content in control subjects ranged between 43.29 and 108.08 pg/ml, mean 84.87 ± 16.17 pg/ml. In T2DM patients, 8-OHdG content of oxidative damage of DNA was greater than controls, 178.35 ± 26.23 pg/ml (range134.55–230.28 pg/ml, *p* = *0.001*). In non-diabetic patients, 8-OHdG content was also significantly higher than in controls 126.78 ± 27.91 pg/ml for oxidative DNA damage (*p* = *0.001*). In addition, there was a significant difference observed between serum 8-OHdG level in T2DM patients compared to non-diabetics (*p* = *0.001*) (Fig. 3).

### Serum 8-OHdG levels in T2DM and non-diabetic with positive *H. pylori* infection

Serum 8-OHdG level in T2DM patients with positive *H. pylori* infection was 188.13 ± 25.40 pg/ml, a significant difference compared to non-diabetics with positive *H. pylori* infection 148.07 ± 19.55 pg/ml (*p* = 0.001) (Table 2). Furthermore, in T2DM group, serum 8-OHdG level was higher with positive *H. pylori* infection 188.13 ± 25.40 pg/ml than in negative *H.pylori* infection 159.38 ± 15.40 pg/ml (*p* = *0.001*) shown in Table 2.

**Table 2:** Distributions of 8-OHdG and Ox-LDL levels in diabetic and non- diabetic patients with (+Ve) *H. pylori* or (−Ve) *H. pylori* infection

Mean ± SD						
	Healthy (n = 50)	Non-diabetic (n = 50)		Diabetic (n = 50)		*P value*
	−ve *H. pylori*	−ve *H. Pylori* (n = 21)	+ve *H. Pylori* (n = 29)	−ve *H. Pylori* (n = 17)	+ve *H. Pylori* (n = 33)	
8-OHdG (pg/ml)	84.87 ± 16.17	98.65 ± 9.47	148.07 ± 19.55	159.38 ± 15.40	188.13 ± 25.40	*0.001* ^*^
Ox-LDL (U/dL)	24.70 ± 12.36	37.68 ± 15.53	72.31 ± 33.80	85.68 ± 35.69	116.79 ± 30.29	*0.001* ^*^

* *p* < *0.05* is considered significant

### Correlation between OxLDL and HbA1c in T2DM and non-diabetic infected with *H. pylori*

**Fig. 4:**
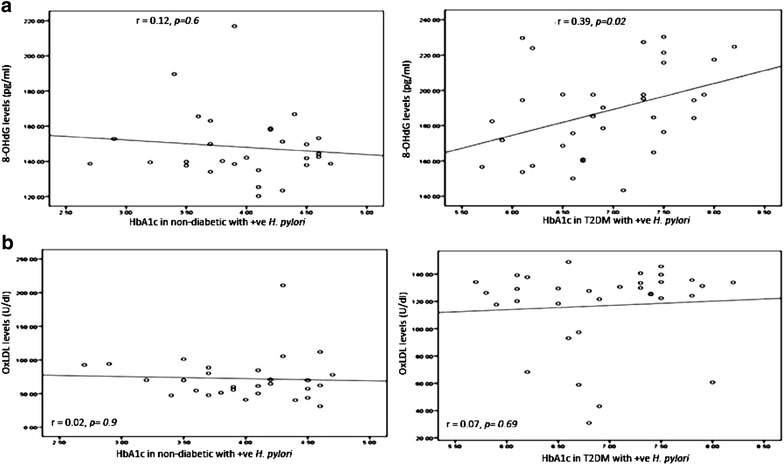
Correlation between HbA1c in non-diabetic and diabetic patients with positive *H. pylori* (expressed in  %) and **a** DNA damage 8-OHdG (expressed in pg/ml). **b** Oxidized LDL (expressed in U/dL). The levels of 8-OHdG or Ox-LDL (*y axis*) were correlated with those of HbA1c (*x axis*). An association was observed between HbA1c in diabetic patients with positive *H. pylori* and 8-OHdG (r = 0.39, *p* = *0.02*). No association was observed between HbA1c in non-diabetic and diabetic patients with positive *H. pylori* and Ox-LDL. Based on the simple linear regression of cases with positive *H. pylori* in non-diabetic and diabetic patients

We observed a correlation between 8-OHdG concentration and HbA1c in T2DM patients infected with *H. pylori* (r = 0.39, *p* = *0.02*). On the other hand, no correlation was observed between 8-OHdG concentration and HbA1c in non-diabetic patients infected with *H. pylori* (r = 0.12*, p* = *0.6*); this correlation is shown in Fig. 4.

### Distribution of serum Ox-LDL

**Fig. 5:**
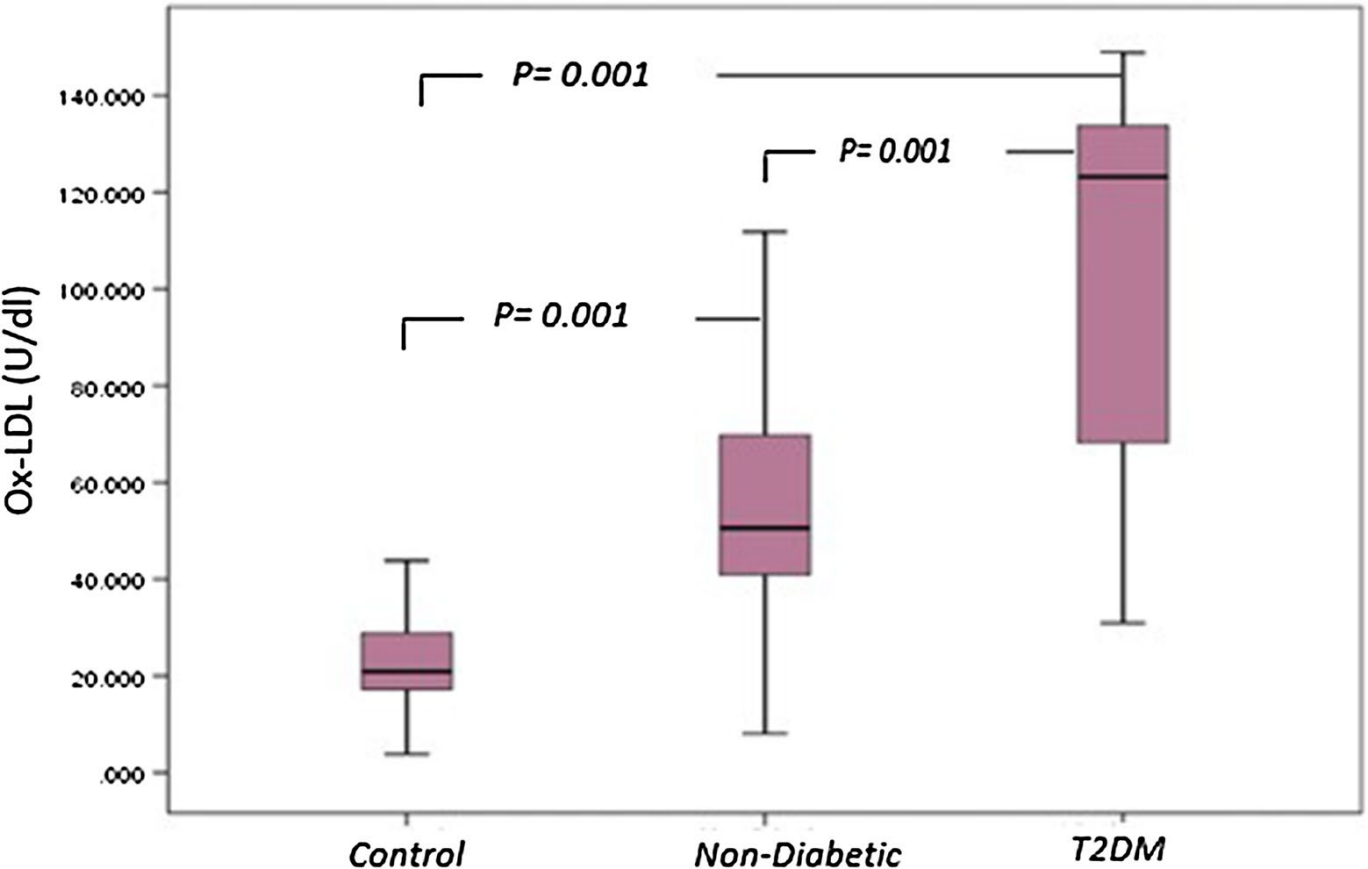
*Box plot* for oxidized LDL in non-diabetic, diabetic patients and control. The *box* represents the interquartile e range. The *whiskers* indicate the highest and lowest values, and the *line* across the *box* indicates the median value. Overall significance of differences between non-diabetic and diabetic group was determined by by 1-way ANOVA

Normal circulating levels of OxLDL level as determined in normal volunteers subjects ranged between 3.81 and 69.12 U/dL, mean 24.70 ± 12.36 U/dL. Serum Ox-LDL levels in patients with T2DM 106.21 ± 35.1 U/dL were significantly higher than in control subjects (*p* = *0.001*). In non-diabetic patients, serum OxLDL level (57.76 ± 32.1 U/dL) was also significantly higher than in controls for oxidation of LDL (*p* = *0.001*). In addition, there was a significant difference observed between Serum Ox-LDL levels in T2DM patients compared to non-diabetics (*p* = *0.001*) (Fig. 5).

### Serum Ox-LDL levels in T2DM and non-diabetic with positive *H. pylori* infection

Serum OxLDL level in T2DM with positive *H. pylori* infection was 116.79 ± 30.29 U/dL, a significant difference compared to diabetics with negative *H. pylori* infection 85.68 ± 35.69 U/dL or in non-diabetics with positive *H. pylori* infection 72.31 ± 33.80 U/dL (*p* = *0.001*) (Table 2).

### Correlation between OxLDL and HbA1c in T2DM and non-diabetic infected with H. pylori

There was no correlation between OxLDL concentration and HbA1c either in T2DM patients infected with *H. pylori* (r = 0.07, *p* = *0.69*) or in non-diabetic patients infected with *H. pylori* (r = 0.02, *p* = *0.9*), this correlation was shown in Fig. 4.

## Discussion

The link between *H. pylori* infection and diabetes remains controversial. Some studies indicated a higher prevalence of infection in diabetic patients [10, 11], and other studies reported no difference [12, 13]. Previous reports, based on serologic antibody detection, have found a high prevalence of *H. pylori* infection among diabetics as compared to the general population [34–36]. The prevalence of *H. pylori* infection ranged between 30 and 80 % in previously reported studies [34, 37].

There are several lines of evidence to implicate increased susceptibility to infection in diabetic patients, where the mechanisms underlying the pathogenesis of diabetes are complex, involving insulin resistance, chronic inflammation, insulin secretion deficiency as a result of pancreas β-cell dysfunction, glucotoxicity, and lipotoxicity [14].

In our study, there was a significant difference in the prevalence of *H. pylori* between cases and controls (*p* = *0.001*), using serological method for diagnosis of *H. pylori* infection. The results therefore showed that *H. pylori* infection is significantly associated with T2DM in our study population. Moreover, there was a significant association between the infection incidence of *H. pylori* among T2DM patients compared to non-diabetic; 66 % (33/50) and 58 % (29/50) respectively (*p* = *0.001*). No correlation was seen between the level of the *H. pylori* IgG antibody and HbA1c % in both groups, T2DM and non-diabetic. Accordingly, no association instigated between prolonged high levels of glucose and the presence of *H. pylori* infection. The relationship between the levels of the *H. pylori* IgG antibody and HbA1c was not clear due to methodological pitfalls in other studies [3]. These pitfalls were selection of inappropriate or insufficient methods used for detecting of *H. pylori* infection and due to population differences, among different studies.

The prevalence of *H. pylori* infection in diabetic patients was different in previous reports. Controversial results of prevalence rates may be related to the epidemiological distribution of infection, nonhomogeneous patient groups or the kind of diagnostic method to detect infection. A seroprevalence study performed in Netherland reported that the frequency of *H. pylori* infection was higher in diabetic patients in comparison with the control subjects [38]. Another seroprevalence study in United Arab Emirates showed that positive antibody titer for *H. pylori* infection (IgG > 300) in diabetics was 76.7 % compared to non-diabetics 64.8 % [38].

In contrast, other studies that showed no association between T2DM and *H. pylori* infection. In a seroprevalence study frequency of *H. pylori* infection was 33 and 32 %, in patients with diabetes and controls respectively [39]. Demir et al. showed that the prevalence of *H. pylori* infection was 61.7 and 58.5 %, among T2DM and non-diabetics respectively [40].

Several hypotheses were presented to confirm the higher prevalence of *H. pylori* infection in diabetic patients such as insulin resistance and abnormal insulin secretion were central to the development of T2DM. One of these hypothesis confirm that; *H. pylori* infection brings about chronic low grade inflammation with up regulation of several cytokines such as CRP, TNF and interleukin (IL)-1β, which may influence insulin action and pancreatic β cell secretion. The other one; *H. pylori*-induced gastritis can potentially affect the secretion of gastric hormones, including leptin, ghrelin, gastrin, and somatostatin, which could affect insulin sensitivity and glucose homeostasis. In addition, other mechanisms and mediators may be involved in the possible causative relationship between *H. pylori* infection and T2DM [14].

Oxidative stress associated with the production of ROS has been shown to play an important role in the pathogenesis of diabetes [41] and *H. pylori* infection [42]. Excess ROS would accelerate oxidative damage to DNA and to other macromolecules, such as proteins and lipids. The present study is one of the few studies which investigated the relationship between Ox-LDL and 8-OHdG, a marker of systemic oxidative stress with *H. pylori*-positive in T2DM patients.

Our data demonstrated that T2DM patients with positive *H. pylori* infection had much higher levels of serum Ox-LDL (116.79 ± 30.29 U/dL) compared with their respective controls. We found no previous reported data linking between OxLDL and T2DM combined with *H. pylori* infection. However, Koichi Ono [43] showed Ox-LDL levels were significantly higher in diabetic patients (n = 30) than in control patients. While, Kayo et al. reported no difference in Ox-LDL levels among patients with *H. pylori* infection and control subjects [24].

Several studies demonstrated that Ox-LDL is a key factor in the initiation and progression of atherosclerosis [44]. Recently, positive associations between chronic *H.pylori* infection and coronary heart disease has been reported [45]; other studies demonstrated that DM considered a risk factor for atherosclerosis and asymptomatic low grade inflammation occurs prior to unconcealed vascular lesions [28]. According to these hypotheses, atherosclerosis is considered a process involving the interplay of inflammation and oxidative stress.

Our data also indicated that there was a significant difference observed between serum Ox-LDL levels in T2DM patients with positive *H. pylori* infection compared to non-diabetics with positive *H. pylori* infection. Our result is supported by the report of Toshima et al. which indicated a significant increase in plasma Ox-LDL in diabetics [46]. In addition, Koichi Ono has shown increased susceptibility of LDL to oxidation in diabetics [43]. Although, there were other reports indicating no increase in Ox-LDL in diabetics [47, 48]. A potential explanation for these discrepant results lies in the hypothesis that patients with DM are more predisposed to infections and severe diseases because of cellular immunity disorders and phagocyte dysfunction caused by hyperglycemia and decreased vascularization. Therefore, patients with DM accompanied with *H. pylori* infection support the concept that oxidative stress associated with neutrophil accumulation and activation plays a role in the inflammatory process [24]. Myeloperoxidase (MPO), a strong pro-oxidant enzyme released from activated neutrophils, has been found to be capable of oxidizing LDL [49]. These hypotheses suggest the possibility that MPO secreted from activated neutrophils in *H. pylori*-mediated gastritis lesions may induce LDL oxidation.

There was no correlation between serum Ox-LDL levels and HbA1c in T2DM (r = 0.07, *p* = *0.69*) and non-diabetic (r = 0.02, *p* = *0.9*) patients with positive *H. pylori*. Koichi Ono and Toshima et al. also found no correlation between plasma Ox-LDL and HbA1c levels in diabetic patients than in non-diabetic patient [43, 46]. Moreover, our data showed no correlation between serum Ox-LDL levels and positive *H. pylori* and negative *H. pylori* infection in diabetic group.

Significantly raised levels of a specific marker of oxidative damage to DNA; 8-OHdG, were reported in *H. pylori* infection [21] and diabetics [32, 50]. As DNA damage is efficiently repaired by cellular enzymes, its measurement gives a snapshot view of the level of oxidative stress, in contrast to measurement of oxidation of other biomolecules which are not repaired and/or have a slow turnover, such as lipids or proteins. DNA oxidation may therefore be of considerable value in following the progress of the disease and its metabolic control [51]. A positive association has been demonstrated between *H. pylori* infection and diabetes with increased oxidative stress. Therefore, our observations showed that diabetic patients combined with *H. pylori* infection have significantly increased oxidative DNA damage. We found a significant increase in serum 8-OHdG level in T2DM (188.13 ± 25.40 pg/mL) and non-diabetic (148.07 ± 19.55 pg/mL) patients with positive *H. pylori* compared to their respective controls (84.87 ± 16.17 pg/mL), confirming the report of Dandong et al. [31].

Moreover, there is greater oxidative DNA damage in T2DM patients with positive *H. pylori* than non-diabetics with positive *H. pylori* infection. Yongsheng Ma et al. [21] showed a significant increase of 8-OHdG in *H. pylori* positive and negative gastric cancer patients. An association between poor glycaemic control in T2DM patients and oxidative stress has been established [40, 52]. This study indicates a correlation between 8-OHdG concentration and HbA1c in T2DM patients infected with *H. pylori* (r = 0.39, *p* = *0.02*). Furthermore, our data showed that hyperglycemia implicated with *H. pylori* infection might lead to increased 8-OHdG level in serum through the overproduction of ROS. This explain why 8-OHdG has been used widely in many studies as endogenous oxidative DNA damage. 8-OHdG can serves as a useful biomarker for the evaluation of oxidative stress in diabetic patients with *H. pylori* infection.

## Conclusion

In summary, the present study suggests that infection *H. pylori* in T2DM was higher compared to non-diabetic population and appears not to be associated with glycemic control; T2DM seems to be associated with increased oxidative stress in *H. pylori* infection. This is the first report as known of a direct association between oxidative DNA damage, serum Ox-LDL levels and T2DM patients companied with positive *H. pylori* infection. Furthermore, 8-OHdG is one of the predominant agents of free-radical-induced oxidative lesions. In our study, we observed that significantly elevated serum Ox-LDL levels in T2DM patients with positive *H. pylori* infection, suggesting hypothesis that high serum level of Ox-LDL levels in T2DM patients with positive *H. pylori* infection considered as a risk factor to atherosclerotic vascular disease and further studies are needed to confirm this hypothesis.

## Abbreviations

*H. pylori*: *Helicobacter pylori*
T2DM: type 2 diabetes mellitus
DM: diabetes mellitus
CRP: C-reactive protein
IL-6: interleukin 6
TNF-α: tumor necrosis factor-α
ROS: reactive oxygen species
Ox-LDL: oxidized low density lipoprotein
AGE: advanced glycation end-products
8-OHdG: 8-hydroxydeoxy guanosine
BMI: body mass index
FBS: fasting blood sugar
PBS: postprandial blood sugar
HbA1c: hemoglobinA1c
DCCT: diabetes control and complications trial
ELISA: enzyme-linked immunosorbent assays
HRP: horseradish peroxidase
TMB: 3,3′,5,5′-tetramethylbenzidine
MPO: myeloperoxidase
